# Selection of food patches by sympatric herbivores in response to concealment and distance from a refuge

**DOI:** 10.1002/ece3.1940

**Published:** 2016-03-22

**Authors:** Miranda M. Crowell, Lisa A. Shipley, Meghan J. Camp, Janet L. Rachlow, Jennifer S. Forbey, Timothy R. Johnson

**Affiliations:** ^1^ School of the EnvironmentWashington State UniversityPullmanWashington; ^2^ Department of Fish and Wildlife SciencesUniversity of IdahoMoscowIdaho; ^3^ Department of Biological SciencesBoise State UniversityBoiseIdaho; ^4^ Department of Statistical ScienceUniversity of IdahoMoscowIdaho

**Keywords:** *Brachylagus idahoensis*, burrow, concealment cover, mountain cottontail, predation risk, pygmy rabbit, sagebrush‐steppe, *Sylvilagus nuttallii*

## Abstract

Small herbivores face risks of predation while foraging and are often forced to trade off food quality for safety. Life history, behaviour, and habitat of predator and prey can influence these trade‐offs. We compared how two sympatric rabbits (pygmy rabbit, *Brachylagus idahoensis*; mountain cottontail, *Sylvilagus nuttallii*) that differ in size, use of burrows, and habitat specialization in the sagebrush‐steppe of western North America respond to amount and orientation of concealment cover and proximity to burrow refuges when selecting food patches. We predicted that both rabbit species would prefer food patches that offered greater concealment and food patches that were closer to burrow refuges. However, because pygmy rabbits are small, obligate burrowers that are restricted to sagebrush habitats, we predicted that they would show stronger preferences for greater cover, orientation of concealment, and patches closer to burrow refuges. We offered two food patches to individuals of each species during three experiments that either varied in the amount of concealment cover, orientation of concealment cover, or distance from a burrow refuge. Both species preferred food patches that offered greater concealment, but pygmy rabbits generally preferred terrestrial and mountain cottontails preferred aerial concealment. Only pygmy rabbits preferred food patches closer to their burrow refuge. Different responses to concealment and proximity to burrow refuges by the two species likely reflect differences in perceived predation risks. Because terrestrial predators are able to dig for prey in burrows, animals like pygmy rabbits that rely on burrow refuges might select food patches based more on terrestrial concealment. In contrast, larger habitat generalists that do not rely on burrow refuges, like mountain cottontails, might trade off terrestrial concealment for visibility to detect approaching terrestrial predators. This study suggests that body size and evolutionary adaptations for using habitat, even in closely related species, might influence anti‐predator behaviors in prey species.

## Introduction

While foraging, small mammalian herbivores face a variety of risks that can affect the value of food patches. Some risks are inherent in the food itself, such as plant fiber and toxins (i.e., plant secondary metabolites; Belovsky and Schmitz 1994; Dearing et al. 2000). Other risks (or costs), such as thermal extremes and predation, might be external to the food patch, but might interact with food quality (Dearing et al. 2008; McArthur et al. 2012, 2014). Therefore, herbivores must weigh the perceived risks of food patches as they choose when and where to forage. When animals perceive higher risks of predation, they might forage in less nutritious but safer patches, or spend less time foraging and more time being vigilant (Rachlow and Bowyer 1998; Altendorf et al. 2001; Hernández and Laundré 2005; Ale and Brown 2009) or hiding (Ydenberg and Dill 1986; Lima and Dill 1990; Alldredge et al. 1991), which could reduce their fitness by reducing nutrient and energy intake. For example, common brushtail possums (*Trichosurus vulpecula*) selected food patches that minimized predation risk when plant toxicity was low, but when plant toxicity increased, possums selected food patches without toxins, but with higher predation risk (Nersesian et al. 2011; Mella et al. 2015).

To minimize the risk of predation, animals might choose food patches that provide higher levels of concealment cover, or provide effective escape cover, such as proximity to refuges (e.g., nests or burrows). For example, common brushtail possums (Nersesian et al. 2012) and European rabbits (*Oryctolagus cuniculus*; Banks et al. 1999) selected food patches with higher concealment or patches closer to a structural refuge when predators or predator cues were present. Therefore, central‐place foragers (e.g., European rabbits, Bakker et al. 2005; American pika, *Ochotona princeps*, Huntly et al. 1986) often experience a gradient of vegetative cover at increasing distances away from their refuge that reflects an increase in predation risk. Although concealment cover is often measured as the percentage of an animal hidden from view from a certain distance and height (Morris 1979; Redmond et al. 1982; Griffith and Youtie 1988; Collins and Becker 2001; Glen et al. 2010; Puan et al. 2011), the way that animals perceive predation risk in relation to concealment cover is likely more complex. Some species, such as song thrushes (*Turdus philomelos*, Götmark et al. 1995) and Townsend's ground squirrels (*Urocitellus townsendii*, Schooley et al. 1996) select intermediate or low levels of concealment cover, possibly because areas with lower levels of concealment have more sightlines, allowing them to detect, and potentially escape, predators sooner or more easily (Embar et al. 2011; Camp et al. 2013).

Physical and physiological characteristics of prey and predators also can influence both actual and perceived predation risk in food patches. The method in which a prey species chooses to avoid approaching predators (e.g., hide or flee) depends not only on concealment cover and distance to the nearest refuge, but also its own size, camouflage, and mobility (Alldredge et al. 1991; Vásquez 1996), and the predator's speed, distance from the prey's current location, how it hunts (i.e., aerial vs. terrestrial, ambush vs. pursuit), when it hunts (i.e., nocturnal vs. diurnal; Ydenberg and Dill 1986), and characteristics of escape terrain and substrate (Kotler et al. 2001). Because of trade‐offs in concealment and the ability to visually detect predators (Camp et al. 2012), and different hunting strategies of predators, the orientation of concealment cover might be as important to prey as the amount of concealment cover. How concealment is arranged in a foraging patch (e.g., terrestrial or aerial) may alter the sightlines that prey species can use to detect approaching predators or that predators may use to detect prey species (Embar et al. 2011). Terrestrial concealment refers to concealment cover that blocks horizontal sightlines along the ground, such as those from a terrestrial predator hunting terrestrial prey. Aerial concealment refers to concealment cover that blocks vertical sightlines from the air looking down, such as those from a perched or flying avian predator hunting terrestrial prey (Camp et al. 2012, 2013). For example, European rabbits in Spain fed closer to more concealed patches during the day, possibly to hide from diurnal avian predators, but at night foraged farther from more concealed patches that might hide nocturnal, terrestrial predators (Moreno et al. 1996). In addition, red‐crested cardinals (*Paroaria coronata*) selected for higher aerial concealment above their nest rather than terrestrial concealment around the sides of their nest (Segura et al. 2012). Aerial concealment, which protects against avian predators, predicted survival of mallard (*Anas platyrhynchos*) nests and chicks better than terrestrial concealment (Guyn and Clark 1997), presumably because their main predators were raptors rather than terrestrial mammalian predators that often use olfactory rather than visual cues when hunting (Conover et al. 2010).

Sympatric species are often confronted with the same habitat conditions and predators, but their life history and physical adaptations that influence their risk of predation might cause them to use the landscape differently (e.g., have different “landscapes of fear”; Brown et al. 1999; Laundré et al. 2001). For example, smaller herbivores may have a wider range of predators and therefore might respond more intensely to perceived risk of predation, including selecting for concealment cover and using refuges for escape. Alternatively, larger herbivores might respond more intensely to predation risk because they are more conspicuous than smaller animals. In addition, because habitat generalists are adapted for a wider variety of habitat conditions, they might respond less intensely to the arrangement of concealment cover than would specialists.

**Figure 1:**
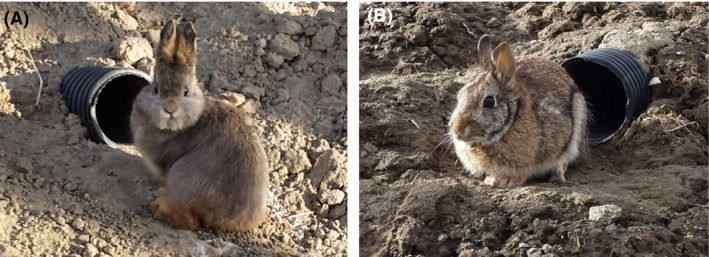
Rabbit species used in our experiments included (A) pygmy rabbit (*Brachylagus idahoensis*) in front of an 8‐cm diameter plastic tube, and (B) mountain cottontail (*Sylvilagus nuttallii*) in front of a 10‐cm diameter plastic tube. Plastic tubes were used as artificial burrow refuges in experiments.

We used a set of controlled foraging trials to compare the response of two species of leporids, pygmy rabbits (*Brachylagus idahoensis*; Fig. 1A) and mountain cottontails (*Sylvilagus nuttallii*; Fig. 1B) to the amount and orientation of concealment cover and distance to a burrow refuge when selecting and exploiting food patches. Pygmy rabbits and mountain cottontails often coexist in sagebrush‐steppe landscapes in the Great Basin of North America (Orr 1940; Chapman 1975; Wilde 1978; Green and Flinders 1980; Thines et al. 2004), yet differ in their size and adaptations to habitat. Pygmy rabbits are the smallest North American leporid (~400 g) and are considered habitat specialists because they rely on sagebrush (*Artemisia* spp.) for food and cover year‐round (Thines et al. 2004; Shipley et al. 2006; Camp et al. 2012). They are also obligate burrowers that require deep soils where they dig natal (Rachlow et al. 2005) and residential burrows as refuges from predators and thermal stress (Green and Flinders 1980; Katzner et al. 1997; Camp et al. 2012; Wilson et al. 2012). In contrast, mountain cottontails are more than twice as large (~1100 g), and are considered habitat generalists because they inhabit a range of habitats from woody, brushy areas, to rocky sagebrush areas, to grassy hills, canyons, and agricultural areas (Chapman 1975). They consume a wide variety of plants (MacCracken and Hansen 1984) and will use burrows, but do not typically dig or require them (Orr 1940; Chapman 1975; Wilde 1978; Green and Flinders 1980; Thines et al. 2004). Both pygmy rabbits, and cottontail spp. experience high annual mortality (i.e., >60%) from both aerial (primarily raptors, ~31% of total known) and terrestrial (primarily coyotes, *Canis latrans*; American badgers, *Taxidea taxus*; weasels, *Mustela* spp., ~33% of total known) predators (Cox et al. 1997; Bond et al. 2001a; Estes‐Zumpf and Rachlow 2009; Crawford et al. 2010). Like many mammalian herbivores, pygmy rabbits and mountain cottontails are expected to respond strongly to the landscape of fear and use a variety of tactics to avoid predators, depending on which predator they perceive as the greatest risk (Shi et al. 1998; Wirsing et al. 2010). We expected both rabbit species to prefer food patches with greater total concealment cover and closer to burrow refuges, but that preference would be stronger for the smaller, obligate burrower, the pygmy rabbit, than the larger mountain cottontail. In addition, we expected that increased concealment cover in patches would reduce preference for closer food patches for both species. Alternatively, because the larger mountain cottontail might be more conspicuous to predators and less likely to use burrows, they might have a stronger preference for total concealment cover. We also expected that pygmy rabbits might be more sensitive to the orientation of concealment cover (i.e., terrestrial vs. aerial vs. random) than would mountain cottontails because they are evolutionarily adapted to sagebrush habitats that generally provide greater and more consistent levels of concealment cover than the gradient of habitats in which mountain cottontails have evolved.

## Materials and Methods

To examine preference for food patches in relation to the (1) amount and (2) orientation of concealment cover and (3) distance to a refuge, we conducted three double choice experiments with five to 11 captive pygmy rabbits (406.7 g ± 7.2) captured in Idaho (Idaho Department of Fish and Game Scientific Collection Permits #100310 and #010813) and Montana (Montana Department of Fish, Wildlife and Parks Scientific Collection Permit #2014‐062) and six to nine captive mountain cottontails (1055.6 g ± 10.2) that had been captured in Washington (Washington Department of Fish and Wildlife Scientific Collection Permit #14‐206). All animals had been in captivity from 2 months to 2 years before experiments began. When rabbits were not being used in experimental trials, they were housed indoors in the Small Mammal Research Facility at Washington State University (WSU), Pullman, Washington, USA with an artificial burrow made of 120‐cm long plastic tube (8‐cm diameter) and an insulated nest box for refuge. Husbandry practices and experimental procedures were approved by the WSU Institutional Animal Use and Care Committee (SOP #4219, ASAF #4398).

**Figure 2:**
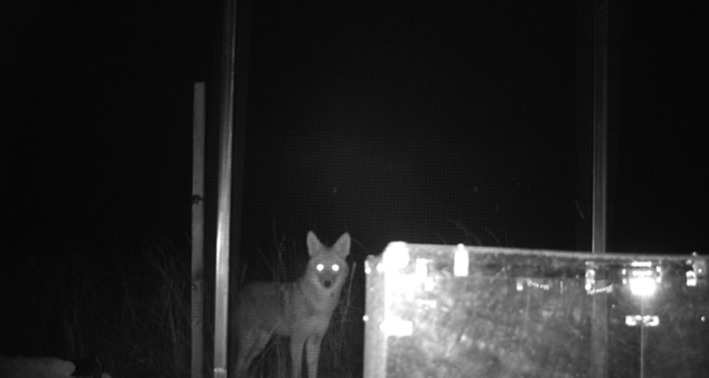
A free‐ranging coyote (*Canis latrans*) looking into the outdoor experimental arenas used in experiment 3, to examine patch choice by pygmy rabbits (*Brachylagus idahoensis*) and mountain cottontails (*Sylvilagus nuttallii*). Rabbits were never at risk of mortality in these experiments, but we controlled for behavioral variation from exposure to natural predator cues, such as the sight, smell, or sound of this coyote, by randomizing the order in which individual rabbits received treatments in each of the feeding experiments.

In each set of experiments, rabbits were offered two food patches in outdoor experimental arenas that were exposed to the sight, smell, and sound of several naturally occurring predators, including coyotes (Fig. 2), American badgers, great‐horned owls (*Bubo virginianus*), and red‐tailed hawks (*Buteo jamaicensis*). Although rabbits in these experiments were never at risk of mortality, we controlled for behavioral variation from exposure to these natural predator cues outside the experimental arenas across the day and season by randomizing the order in which individual rabbits received treatments for each of the feeding experiments. Each patch contained a bowl of ad libitum (i.e., 50 g for pygmy rabbits, 70 g for mountain cottontails) rabbit pellets (Purina^®^ Rabbit Chow Professional; Purina Mills, LLC., St. Louis, MO) placed under a 0.46 × 0.46 × 0.46 m clear acrylic box with a 10‐cm diameter opening. Either the amount or orientation of concealment cover, or the distance to a refuge, was varied between patches in each trial. We recorded the amount of food offered and remaining (orts) after 24 h for each patch in each experiment (encompassing both diurnal and nocturnal intake), and corrected for dry matter by drying the orts and a sample of the food pellets offered at 100°C for ≥24 h.

### Experiment 1: total amount of concealment cover

**Figure 3:**
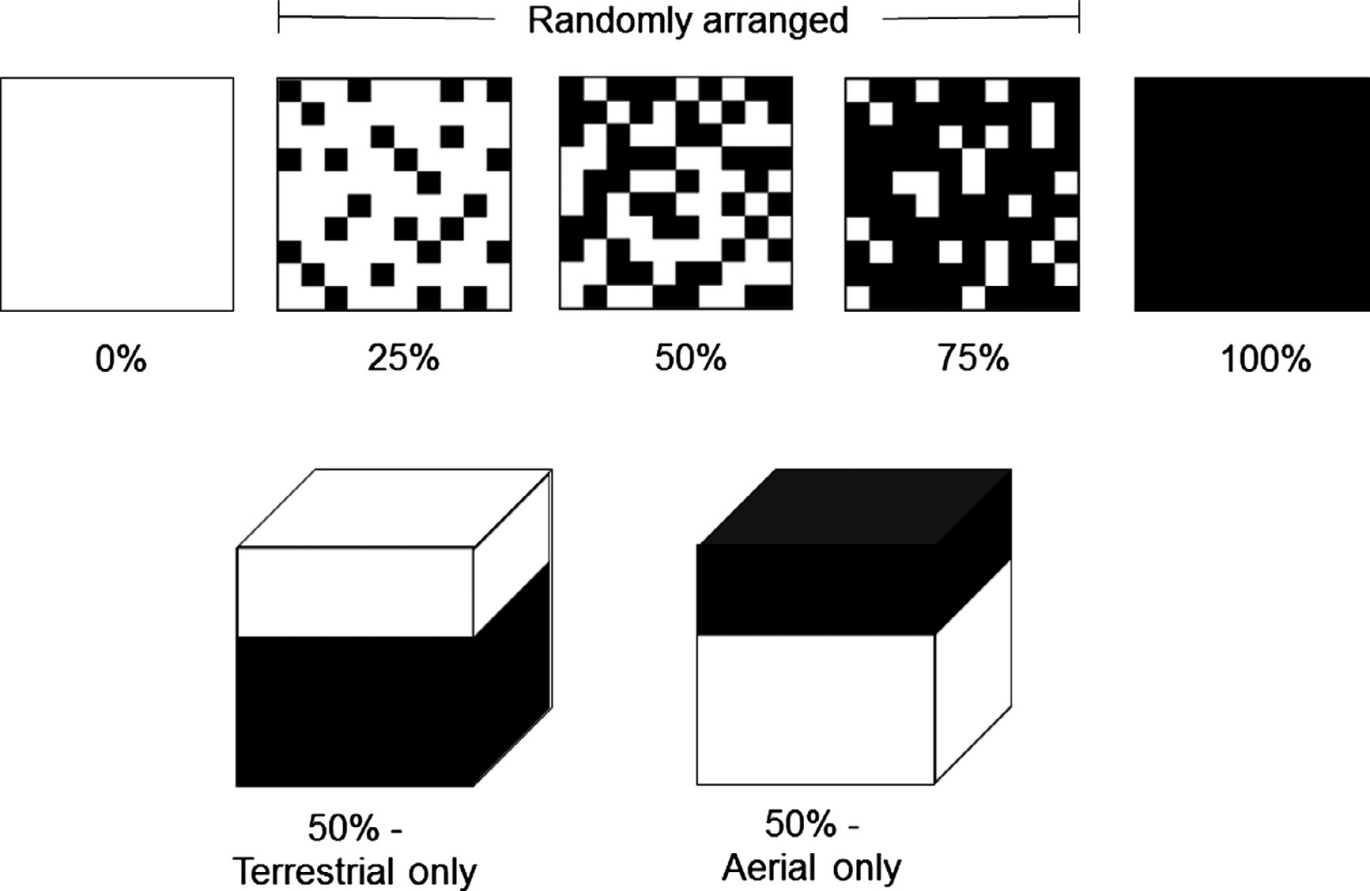
Transparencies placed on the five sides of clear acrylic boxes to create feeding patches that varied in the total amount or orientation of concealment cover. Areas shown in black were opaque, and areas shown in white were transparent, and squares within panels were randomly arranged. For 0%, 25%, 50%, 75%, and 100% concealment cover, the top panel was identical to the four side panels. For the terrestrial concealment, the top panel and upper 17 cm of the side panels were completely transparent, and the lower 27 cm of the side panels were opaque black. For aerial concealment, the top panel and upper 17 cm of the side panels were opaque black.

We compared preference for food patches with 0%, 25%, 50%, 75%, and 100% total concealment cover between rabbit species by conducting these choice experiments in nine outdoor arenas (~3.8 × 3.6 m). Each arena contained two familiar refuges, an insulated nest box placed on one side of the arena, and an artificial burrow made of 120‐cm long plastic tube (8‐cm diameter) for pygmy rabbits, or a wooden hutch for cottontails, placed on the opposite side of the arena. Concealment cover was varied by attaching a transparency sheet to each of the five sides of a clear acrylic box that was placed over each food patch. Each transparency was divided into 100 squares (0.46 × 0.46 cm). To create different levels of concealment, randomly selected squares were colored an opaque black (Fig. 3). Each rabbit completed 10 choice trials with each pairwise combination of concealment cover at food patches placed an equal distance (1.5 m) from the nest box. We selected this distance because a previous field experiment found that pygmy rabbits fled to a burrow refuge 70% of the time they perceived an approaching risk when they were within 1 m of these burrow refuges (Camp et al. 2012). These experiments were conducted during May, August and September 2013 and July 2014. Trials with pygmy rabbits and mountain cottontails were conducted simultaneously to control for changing weather and moon phase. Each pairwise combination was assigned a number and order was determined with a random number generator. The location of the patch (left or right side of the arena) was determined by a coin flip.

### Experiment 2: orientation of concealment cover

We compared preference for food patches in relation to the orientation of concealment cover by creating two new types of concealment cover transparencies with a total of 50% cover, but arranged with either opaque black (100%) cover only around the bottom 29 cm of the four sides of the box (i.e., only terrestrial cover that would provide concealment from terrestrial predators), or opaque black (100%) cover only over the top surface of the acrylic box and the top 17 cm of the four sides of the box (i.e., only aerial cover that would provide concealment from avian predators; Fig. 3). After a preliminary trial exposing rabbits to all types (levels and orientation) of concealment cover, each rabbit completed seven double choice trials with each pairwise combination of concealment cover that included terrestrial or aerial treatments, including 50% of the total area oriented only terrestrially, 50% oriented only aerially, 50% arranged randomly throughout the box, 100% (entire box with opaque black cover) and 0% (entire box transparent) at food patches placed an equal distance (1.5 m) from the nest box, using the methods described previously for the amount of total concealment cover experiments. These experiments were conducted simultaneously for pygmy rabbits and mountain cottontails between January and April 2014, in nine outdoor arenas. Each pairwise combination was assigned a number and order was determined with a random number generator. The location of the patch (left or right side of the arena) was determined by a coin flip.

### Experiment 3: distance from a refuge

We compared preference for food patches in relation to distance from a burrow refuge by conducting a series of foraging trials with food patches placed at three different distances (i.e., 1.5 m = close, 5 m = moderate, 8.5 m = far) from artificial burrow systems located within a 0.5‐m high soil mound (six entrances, 8–10 cm diameters). Artificial burrows were similar to those used by free‐ranging pygmy rabbits and sometimes mountain cottontails, which are typically found on natural soil mounds and have multiple entrances that are 10–12 cm in diameter (Green and Flinders 1980). We conducted these choice trials in three outdoor arenas (~4 × 12.5 m), each with the burrow mound on one end of the arena. A nest box was placed on top of the burrow mound to provide an additional familiar refuge. We repeated these trials four times with cover boxes at four levels of concealment cover that were arranged randomly (0%, 25%, 50% and 100%). In each trial at each concealment level, rabbits were offered paired food patches at two distances from the burrow mound in three distance combinations – close/moderate, moderate/far, and close/far. Because we had only three large arenas for these experiments, trials were conducted with pygmy rabbits from September to November 2013, and with mountain cottontails from March to April 2014. Temperatures and day length were similar during these periods, and both species have been documented to use burrows in all seasons (Orr 1940; Chapman 1975; Thines et al. 2004).

### Data analysis

We first compared total intake (sum of intake from both patches within a choice trial) by species among trials (pairs of treatments) within each of the three experiments to determine if rabbits increased their intake for any concealment or distance combination using a one‐way ANOVA.

To compare proportion of food eaten from paired food patches in relation to the amount of concealment cover, we used a mixed model with main effects of rabbit species, trial type (i.e., concealment combination) and the interaction of species and trial type, with individual rabbit as a random effect (PROC MIXED, Ver. 9.3; SAS Institute Inc. 2008). We used a contrast statement to compare the proportion consumed in the most concealed patch to 0.5 (i.e., equal preference between paired food patches) for each rabbit species. To compare proportion of food consumed from paired food patches in relation to orientation of cover, we used a similar model including species, trial type, and interactions, and used a contrast statement to compare each trial (orientation of concealment combination) with 0.5 for each rabbit species. Finally, to compare the proportion of food consumed from paired food patches in relation to the distance from a burrow refuge, we used a mixed model with main effects of rabbit species, distance combination (i.e., close‐moderate, moderate‐far, close‐far) and total concealment cover (0%, 25%, 50%, and 100%) and all interactions of main effects. We used a contrast statement to compare the proportion consumed in the closest patch to 0.5 for each rabbit species.

## Results

Within an experiment, both pygmy rabbits and mountain cottontails consumed the same total dry mass of pellets (sum of intake from pairs offered simultaneously) across trials. Pygmy rabbits consumed a total mass of pellets per day that averaged 34.8 g (*F*_9,82_ = 0.11, *P* = 0.99, SD = 10.1) for total amount of concealment cover (experiment 1), 30.5 g (*F*_6,36_ = 0.38, *P* = 0.89, SD = 6.0) for orientation of concealment cover (experiment 2), and 32.1 g (*F*_11,60_ = 1.54, *P* = 0.14, SD = 6.5) for distance from a refuge experiments (experiment 3). Mountain cottontails consumed a total mass of pellets per day that averaged 58.6 g (*F*_9,64_ = 1.16, *P* = 0.34, SD = 11.5) for total amount of concealment cover, 61.0 g (*F*_6,56_ = 0.30, *P* = 0.93, SD = 13.9) for orientation of concealment cover, and 68.4 g (*F*_11,60_ = 0.37, *P* = 0.96, SD = 13.0) for distance from a refuge experiments.

### Experiment 1: total amount of concealment cover

**Figure 4:**
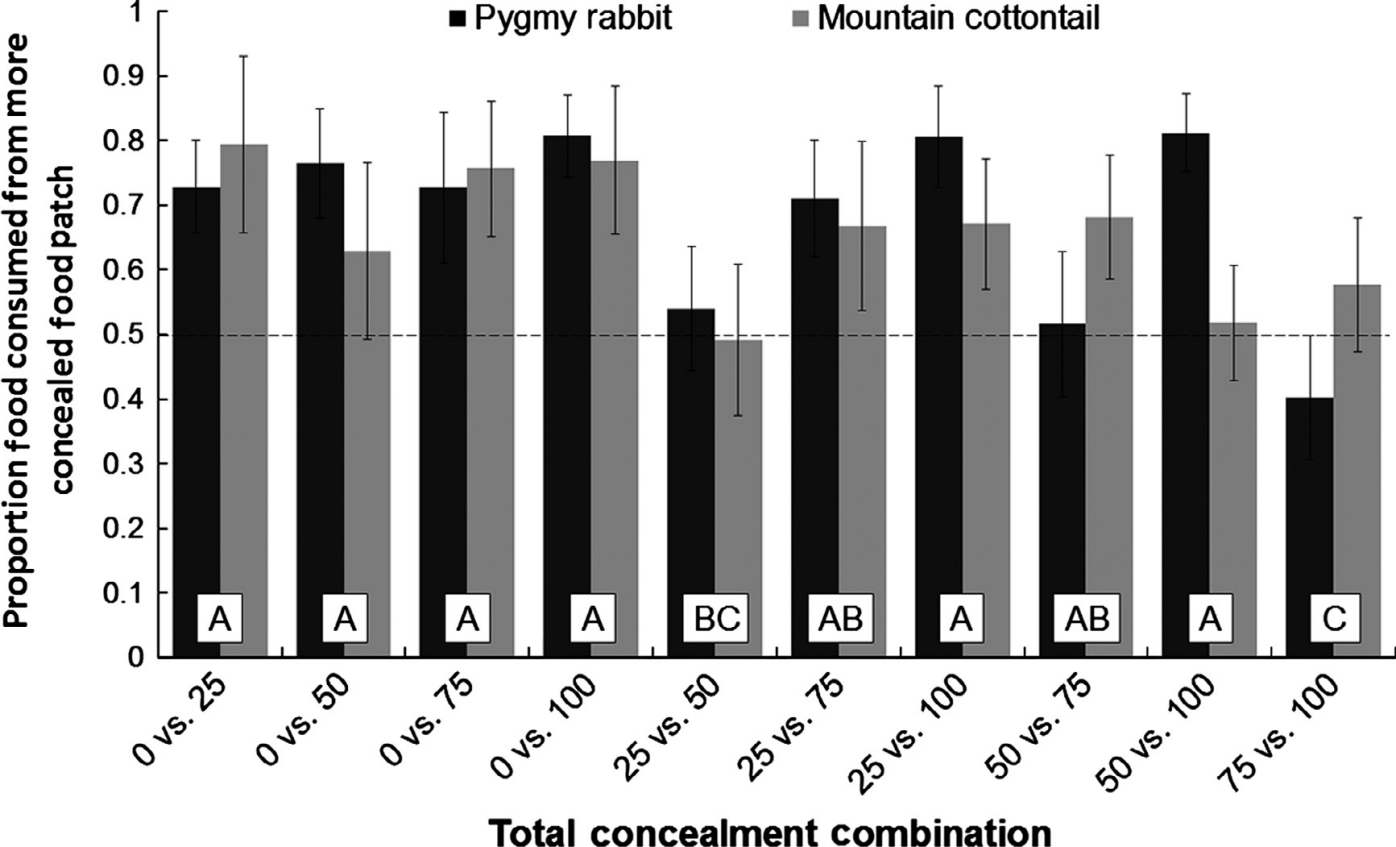
The average proportion of food consumed by pygmy rabbits (*Brachylagus idahoensis*) and mountain cottontails (*Sylvilagus nuttallii*) from the food patch with greater total concealment for each paired concealment cover combination. Both pygmy rabbits and mountain cottontails consumed proportions >0.5 from the most concealed patch across concealment combinations with *α *= 0.05 and different letters denote significant differences in mean proportions among concealment combinations. Pygmy rabbits and mountain cottontails did not differ in proportions consumed from more concealed patches (*P* = 0.56) nor was there a species × concealment combination interaction (*P* = 0.36).

As predicted, both pygmy rabbits and mountain cottontails preferred to forage in patches with greater concealment cover but, contrary with our predictions, they did not differ in the degree to which they preferred greater levels of concealment cover. The proportion of food consumed from the most concealed patch varied with concealment combinations (i.e., trial; *F*_9,127_ = 2.33, *P* = 0.02), but not rabbit species (*F*_1,127_ = 0.34, *P* = 0.56), nor trial × species interaction (*F*_9,127_ = 1.11, *P* = 0.36). Across trials, the proportion consumed from the most concealed patch in each pair by pygmy rabbits (*t*_127_ = 4.44, *P* < 0.0001) and mountain cottontails (*t*_127_ = 3.14, *P* = 0.0021) was >0.5 (Fig. 4). The proportion consumed from the most concealed patch was greatest when the least concealed patch had 0% cover and when the difference between concealment levels was greatest (Fig. 4).

### Experiment 2: orientation of concealment cover

**Figure 5:**
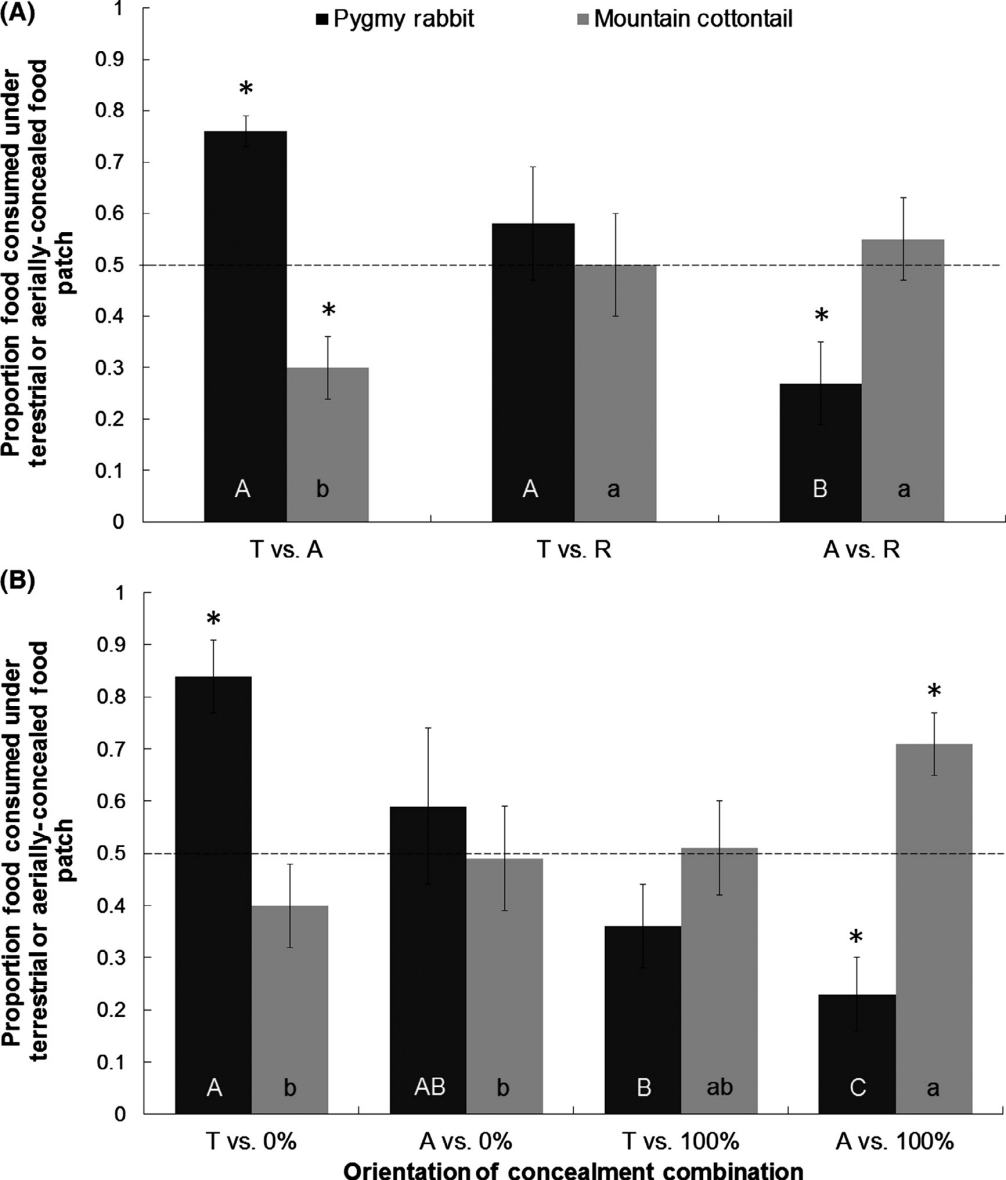
The proportion of food pygmy rabbits (*Brachylagus idahoensis*) and mountain cottontails (*Sylvilagus nuttallii*) consumed from the food patch with 50% terrestrial‐only (T) or aerial‐only (A) concealment cover when paired with (A) another patch with 50% concealment cover arranged in a different orientation (i.e., T, A, or R [50% random cover over entire box]) and (B) another patch with either 0% or 100% concealment. Capital letters denote significant differences in mean proportion consumed among concealment combinations for pygmy rabbits, and lower case letters denote differences for mountain cottontails. An asterisk denotes proportions that were significantly different from 0.5 for each species with *α *= 0.05.

Pygmy rabbits and mountain cottontails differed in their preference for the orientation of concealment cover, but contrary with our predictions, each preferred a different orientation of concealment. The proportion consumed from the terrestrially or aerially concealed food patch did not differ with trial type (i.e., orientation of concealment combination, *F*_6,78_ = 1.33, *P* = 0.25) nor rabbit species (*F*_1,78_ = 0.27, *P* = 0.61), but differed with the trial × species interaction (*F*_6,78_ = 8.13, *P* < 0.0001). When offered choices between 50% total concealment provided in three different orientations (terrestrial‐only, aerial‐only, and random), pygmy rabbits consumed a greater proportion of their daily intake from patches with 50% terrestrial concealment when paired with a patch with 50% aerial concealment, but a lower proportion from food patches with 50% aerial concealment than a food patch with 50% concealment arranged randomly (Fig. 5a). On the other hand, mountain cottontails consumed a greater proportion of food from patches with 50% aerial concealment than 50% terrestrial concealment (Fig. 5a).

The two rabbit species also differed in the proportion consumed from a food patch with either 50% terrestrial or aerial concealment when paired with a food patch with either 0% or 100% concealment cover. Pygmy rabbits consumed a greater proportion of food from patches with terrestrial concealment than from food patches with no concealment (0%), but a lower proportion from food patches with aerial concealment than from food patches with 100% concealment (Fig. 5b). In contrast, mountain cottontails consumed a greater proportion of food from patches with aerial concealment than from food patches with 100% concealment.

### Experiment 3: distance from a refuge

**Figure 6:**
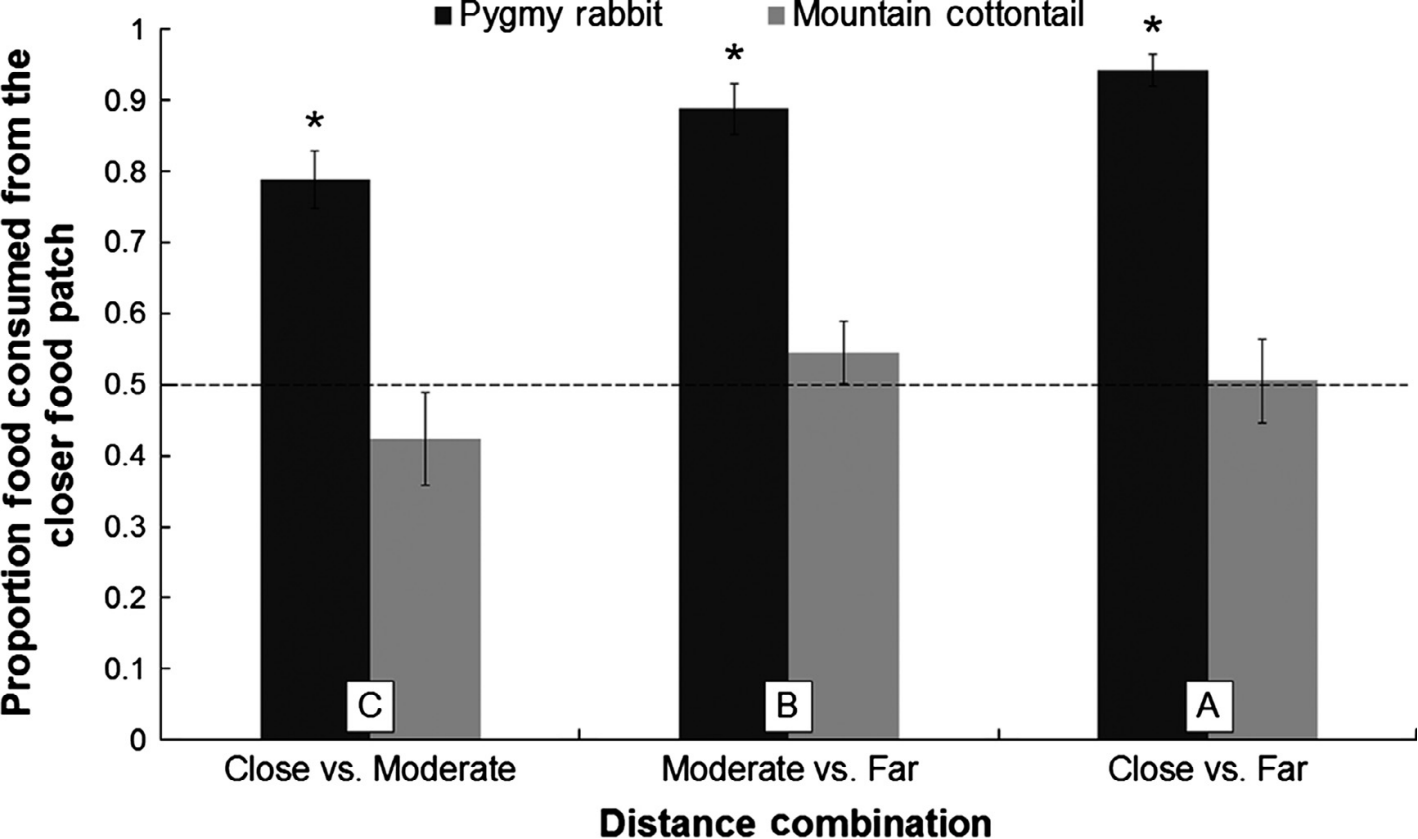
The proportion of food pygmy rabbits (*Brachylagus idahoensis*) and mountain cottontails (*Sylvilagus nuttallii*) consumed from the closer food patch when paired with another patch at close (1.5 m), moderate (5 m) or far (8.5 m) distances from the burrow refuge. Paired patches in the close versus moderate and moderate versus far combinations were 3.5 m apart and patches in the close versus far combination were 7 m apart. Different letters denote significant differences among the main effect of distance combinations across rabbit species (i.e., the distance × species combination was not significant). Asterisks denote mean proportions by species and distance combination that were >0.5 with *α *= 0.05. The proportion consumed in the closer food patch was greater for pygmy rabbits than cottontails for all distance combinations (*P* < 0.0001).

As predicted, pygmy rabbits strongly selected for food patches closer to a burrow refuge, whereas mountain cottontails did not respond to distance from burrows when selecting food patches. The proportion consumed from the closest patch varied with distance combination (*F*_2,110_ = 4.44, *P* = 0.01), and rabbit species (*F*_1,110_ = 29.31, *P* < 0.0001), but not with concealment cover (*F*_3, 110_ = 1.02, *P* = 0.38) or any interaction of main effects (all *P* ≥ 0.16). We found that pygmy rabbits consumed at least 74% of their daily intake from the closer food patch for all distance combinations at all levels of concealment cover (*t*_110_ = 7.95, *P* < 0.0001; Fig. 6), and a greater proportion from the closer food patch when the closer food patch was nearer to the burrow refuge (close rather than moderate) and the food patches were farther apart (3.5 vs. 7 m; Fig. 6). In contrast, mountain cottontails consumed similar proportions of their daily intake from both food patches at all distance combinations, at all levels of concealment (*t*_110_ = 0.29, *P* = 0.77; Fig. 6).

## Discussion

Life history characteristics influenced how two prey species used their habitat to balance their perceived predation risk. When selecting food patches, the smaller habitat specialist (pygmy rabbit), and the larger habitat generalist (mountain cottontail) responded similarly to the amount of concealment from predators, but differently to the orientation of concealment, and distance from a burrow refuge. Pygmy rabbits and mountain cottontails both exhibited a strong preference for feeding under greater concealment cover, but when total concealment was equal, pygmy rabbits preferred patches that offered more terrestrial concealment, whereas mountain cottontails avoided patches with complete terrestrial concealment (i.e., terrestrial‐only or 100%) when patches with aerial‐only concealment were available. Not surprisingly, only the burrow obligate, the pygmy rabbit*,* selected food patches closer to a burrow refuge, even when the next food patch was only 3.5 m away, regardless of the total concealment cover present at the food patches.

These results suggest that although both pygmy rabbits and mountain cottontails may prefer to use total concealment to hide from predators, the two species may perceive different types of predators (terrestrial vs. aerial) as more risky, thus use concealment provided by vegetation differently. In addition, pygmy rabbits likely perceive burrow refuges as less risky than concealment provided by sagebrush outside the burrow, thus, prefer to stay close to burrow refuges while foraging. Hiding in shrubs and in burrow refuges might be more important for reducing predation risk to pygmy rabbits than mountain cottontails because of their smaller size, reduced mobility, specialized coloration, and ability to dig burrows. Because they are less than half the size of mountain cottontails, pygmy rabbits might be more vulnerable to predators, especially smaller‐bodied raptors, such as northern harriers (*Circus cyaneus*) or Cooper's hawks (*Accipiter cooperii*), and small mammals, such as weasels (Cox et al. 1997; Estes‐Zumpf and Rachlow 2009). In addition, some literature suggests that pygmy rabbits might run more slowly than other leporid species (Orr 1940; Wilde 1978; Green and Flinders 1980; Gabler et al. 2001). However, these slower speeds might simply reflect normal behavior of animals near their refuge. For example, free‐ranging pygmy rabbits took longer to flee from their hiding spots when they were better concealed (Camp et al. 2012) and have been observed to sit motionless at the base of sagebrush plants (M.M. Crowell, L.A. Shipley, M.J. Camp, J.L. Rachlow, J.S. Forbey, personal observation). Likewise, to combat the metabolic cost of fleeing from predators (Ydenberg and Dill 1986), woodchucks (*Marmota monax*) fled significantly slower when they were within 2 m of their burrow (Bonenfant and Kramer 1996). In addition, the continuous gray‐brown coat of pygmy rabbits might be better camouflaged by sagebrush plants (Green and Flinders 1980; Stoner et al. 2003), than the white coloration on the undersides and tail of mountain cottontails (Orr 1940; Chapman 1975), which might serve as a warning to other rabbits when fleeing (Smythe 1970; Stoner et al. 2003).

Terrestrial concealment cover may also reduce predation risk of pygmy rabbits more than that of mountain cottontails because of their greater reliance on burrows for refuge. Although burrows provide an effective escape from aerial predators, mammalian predators might be able to enter the burrow (i.e., weasels) or excavate them (i.e., coyotes, badgers; Wilde 1978). Therefore, remaining concealed from terrestrial predators might be more important than from aerial predators when pygmy rabbits are near their burrows. Because mountain cottontails are not believed to create their own burrows or might be too large to use some of the burrows created by sympatric pygmy rabbits, they do not always have access to burrow refuges. Mountain cottontails are more likely to run than to hide when disturbed, and have been documented to run 5–15 m away from a point of danger, and if disturbed again, they will run in a circular path, presumably to confuse the potential threat (Orr 1940; Chapman 1975). In addition, other studies have also documented that eastern cottontails (*Sylvilagus floridanus*), a similar species to mountain cottontails, prefer to forage and rest in or near to areas that offer greater concealment from shrub cover (Chapman 1975; Swihart and Yahner 1984; Bertolino et al. 2011). These observations suggest that burrow refuges might be riskier than shrub cover for mountain cottontails because shrubs might provide more visibility, especially terrestrially, of the surroundings that allow early detection of approaching terrestrial predators, providing time and room to escape (Bond et al. 2001b). Although measuring “visibility” from the animal's perspective is difficult (Boyer et al. 2006; Camp et al. 2013), future studies should examine how habitat features influence both concealment and visibility, and how animals trade off these correlated (Camp et al. 2013), but functionally different, aspects of security cover.

Pygmy rabbits clearly selected food patches closer to their burrow refuges, whereas distance to burrow refuges did not influence food patch selection by mountain cottontails. Our results are consistent with those from another habitat generalist, the eastern cottontail*,* which foraged equally from food patches regardless of the amount of, or distance to, burrow refuges or concealment cover (Smith and Litvaitis 2000). Although increased concealment cover did not reduce the preference of pygmy rabbits for closer food patches in our experiments, concealment cover did influence the use of burrows by free‐ranging individuals. Pygmy rabbits, which often use more than one burrow refuge concurrently (Thines et al. 2004; Sanchez and Rachlow 2008; Wilson et al. 2012), switched among burrow refuges more often and had larger home ranges at sites where greater shrub cover was present across the landscape (Sanchez and Rachlow 2008). In addition, pygmy rabbits exhibited different movement patterns and burrow switching behaviors based on the dispersion of habitat resources (Sanchez and Rachlow 2008). Therefore, when concealment cover is more abundant and evenly distributed across the landscape, pygmy rabbits might be able to forage farther from their burrow refuges and access a wider variety of food choices while remaining relatively concealed from predators (Burak 2006). However, perception of and sensitivity to predation risk, and therefore, selection for cover by free‐ranging animals, may vary with the animal's sex, age, and reproductive status. For example, male pygmy rabbits and eastern cottontails have larger home ranges during the spring breeding season, likely reflecting mate‐searching activities (Bond et al. 2001b; Sanchez and Rachlow 2008). Although limitations in the number of large experimental enclosures precluded comparing preference for patches in relation to distance to burrow refuges simultaneously in pygmy rabbits and mountain cottontails, movement patterns of free‐ranging pygmy rabbits did not differ among seasons (Sanchez and Rachlow 2008) suggesting that concealment cover and food resources influence movement patterns at smaller spatial scales to a greater degree than season. Future research should compare responses to security cover between sympatric rabbit species across seasons.

In niche theory, ecologically similar species must occupy their own unique niches to coexist in a landscape (Pianka 1981). Our study suggests that the mechanisms that allow pygmy rabbits and mountain cottontails to share sagebrush‐steppe landscapes include not only their differential use of food resources (Shipley et al. 2006), but also how they use security cover. Although pygmy rabbits and mountain cottontails both use sagebrush and burrow resources, pygmy rabbits dig and rely on burrow refuges and therefore, they require parts of the sagebrush‐steppe landscape with mounds and deeper soils (Green and Flinders 1980; Weiss and Verts 1984), whereas mountain cottontails usually inhabit rockier areas of sagebrush‐steppe landscapes that are likely less suitable for digging burrow refuges (Chapman 1975). Although not yet demonstrated in field studies, differential preference for orientation of concealment cover also might promote spatial separation of pygmy rabbits and mountain cottontails within sagebrush‐steppe landscapes. Future research could compare selection of concealment in landscapes where these leporids coexist and where they occur independently.

Understanding how sympatric species perceive the amount and type of security features within a landscape (i.e., landscape of fear), helps predict differential use of habitat by herbivores residing in changing landscapes. For example, the sagebrush‐steppe is one of the most imperiled ecosystems in North America because of sustained degradation, fragmentation, and conversion to other land uses (Knick et al. 2003). Therefore, many of the species that inhabit this ecosystem are of conservation concern, including habitat specialists, such as pygmy rabbits (USFWS 2014), greater sage‐grouse (*Centrocercus urophasianus*; Schroeder et al. 2004), sagebrush sparrows (*Artemisiospiza nevadensis*), Brewer's sparrows (*Spizella breweri*; Knick and Rotenberry 2000), and generalists, such as mountain cottontails, American badgers, least chipmunks (*Tamias minimus*), sharp‐tailed grouse (*Tympanuchus phasianellus columbianus*; McDonald and Reese 1998), mule deer (*Odocoileus hemionus*), and elk (*Cervus elaphus*; Lehmkuhl et al. 2001). Because our research suggests that these two leporid species respond differently to the orientation of concealment cover, changes in sagebrush‐steppe landscapes caused by altered fire regimes, invasive species, global climate change, and intensive livestock grazing (Hemstrom et al. 2002) might be expected to affect these species differently, and determine their distribution on the landscape. Therefore, understanding how prey use food and cover resources and how they respond behaviorally to perceived predation risk is critical for managing threatened populations and habitats. For example, habitat and burrow specialists might be more patchily distributed across a landscape because they rely on greater concealment or their burrow refuges to avoid predators. In contrast, habitat generalists that use burrow refuges opportunistically might use landscapes more uniformly or randomly because they rely less on burrow refuges for protection, and more on detecting predators early and fleeing. Habitat and burrow specialization, body size, and predator‐evasion tactics may be only a few of many characteristics of prey animals that influence how they perceive and respond to predation risk. Furthermore, predation risk is only one of the factors herbivores must consider when selecting food patches and resting areas. Examining how animal characteristics influence trade‐offs among predation risk and other risks or resources will increase our ability to assess habitat quality and provide further insight into how foraging animals share resources across landscapes.

## Conflict of Interest

None declared.
